# A Definition of “Multitargeticity”: Identifying Potential Multitarget and Selective Ligands Through a Vector Analysis

**DOI:** 10.3389/fchem.2020.00176

**Published:** 2020-03-13

**Authors:** Juan Francisco Sánchez-Tejeda, Juan F. Sánchez-Ruiz, Juan Rodrigo Salazar, Marco A. Loza-Mejía

**Affiliations:** ^1^Facultad de Ciencias Químicas, Universidad La Salle, Mexico City, Mexico; ^2^Ciencia y Estrategia S.A. de C.V., Mexico City, Mexico

**Keywords:** multitarget drugs, drug discovery, drug-design, multitarget index, multiple sclerosis, polypharmacology

## Abstract

The design of multitarget drugs is an essential area of research in Medicinal Chemistry since they have been proposed as potential therapeutics for the management of complex diseases. However, defining a multitarget drug is not an easy task. In this work, we propose a vector analysis for measuring and defining “multitargeticity.” We developed terms, such as order and force of a ligand, to finally reach two parameters: multitarget indexes 1 and 2. The combination of these two indexes allows discrimination of multitarget drugs. Several training sets were constructed to test the usefulness of the indexes: an experimental training set, with real affinities, a docking training set, within theoretical values, and an extensive database training set. The indexes proved to be useful, as they were used independently *in silico* and experimental data, identifying actual multitarget compounds and even selective ligands in most of the training sets. We then applied these indexes to evaluate a virtual library of potential ligands for targets related to multiple sclerosis, identifying 10 compounds that are likely leads for the development of multitarget drugs based on their *in silico* behavior. With this work, a new milestone is made in the way of defining multitargeticity and in drug design.

## Introduction

In the field of polypharmacology, combinatorial therapies and multitarget drugs are the main alternatives for dealing with complex diseases. The first one consists of a combination of multiple single-targeted drugs. On the other hand, multitarget drugs are molecules with the ability to act on different targets at the same time. Designing multitarget drugs is a problematic task; however, it solves several concerns that are seen in combinatorial therapies, such as complex therapeutic regimens, difficulty in including numerous drugs in a single formulation and drug interactions at the different pharmacokinetics levels: absorption, distribution, metabolism, and elimination (Rosini, 2014). In the last two decades, the number of multitarget drugs on the market has been rising. From 2015 to 2017, 21% of the drugs approved by the Food and Drug Administration (FDA) were multitarget drugs (MTD), primarily antineoplastic agents (Ramsay et al., 2018). This trend may indicate that the number of multitarget drugs will continue to rise since they present advantages over single-target drugs. For example, MTDs have higher *in vivo* efficacy, and several *in silico* methods and strategies for designing them are currently being developed (Zhang et al., 2017). A common strategy is to combine two pharmacophores in the same molecule or partially overlap them, allowing binding to two or more targets (Talevi, 2015).

Binding to two or more targets at the same time offers the possibility of treating multifactorial diseases. Neurodegenerative diseases are a potential field for multitarget drugs. For example, ladostigil is a dual cholinesterase–monoamine oxidase-B (MAO-B) inhibitor currently being researched for the treatment of Alzheimer's disease and other neurodegenerative diseases (van der Schyf, 2011). Cancer is another relatively emergent field for multitarget drugs, mainly as more druggable targets are being discovered. The use of multitarget drugs is promising as it lowers the possibility of the disease to evolve into a drug-resistant phenotype (Xie and Bourne, 2015). Currently, several anti-cancer drugs are considered multitarget drugs since they inhibit two or more kinases or receptors (Lu et al., 2012). Another example is in the field of microbiology, in which dual ligands can be used to treat tuberculosis. This dual mechanism of action is useful in treating multidrug-resistant *Mycobacterium tuberculosis* (Chiarelli et al., 2018).

One of the limitations that multitarget drug design faces is data analysis. In some cases, the number of targets or compounds being analyzed can be high. In PubChem, 71,303 molecules have been identified as ligands that have two or more biological targets, and more than 30,000 ligands were found to be active against more than 400 targets (Hu et al., 2014). Quantifying and defining “multitargeticity” may be useful for analyzing these datasets. Additionally, multitarget metrics could help multitarget drug design by providing comparable and workable parameters for drugs and ligands.

To the best of our knowledge, there is no current measurement of “multitargeticity,” i.e., how multitargeted a ligand is. Construction of a multitarget parameter should not be based only on the simple average of the *in silico* or experimental data; for example, highly selective ligands to a single target would appear as multitarget drugs, since the average is a measure sensitive to extreme values. With this in mind, our research group suggested the use of a virtual multitarget parameter, which consisted of a weighted average of the docking scores of potential biopesticides (Loza-Mejía et al., 2018). This analysis proved useful for comparing a ligand's “multitargeticity.” However, a more rigid index may help even further in multitarget drug design.

**Figure 1:**
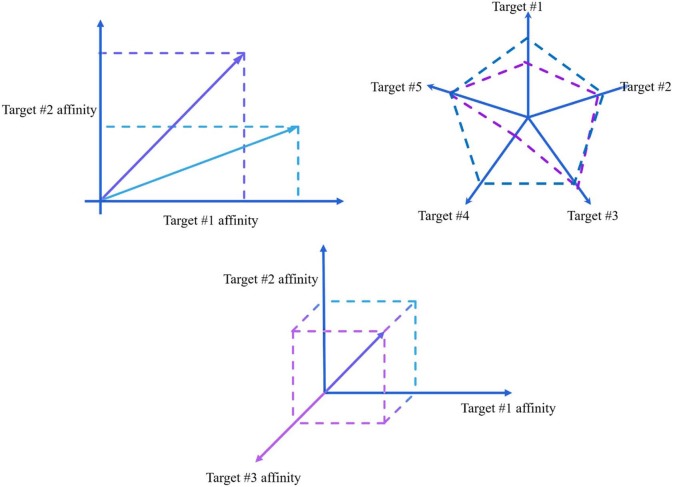
Plotting the ligands' affinities in a dispersion plot describe vectors in a 2D space. For further targets, in a radial plot, an ideal multitarget would describe a regular polygon. Measuring the area of these polygons is influenced by the order in which the targets are plotted. A hypercube is not affected by ordering and therefore makes an ideal representation of what a multitarget ligand would be.

Originally, this project started with the purpose of designing dual ligands. We designed 211 ligands, and we wanted a parameter that could summarize or identify the ligands that had the most potency toward the two targets (the nature of the ligands and the targets will be explained later). To analyze the data, we plotted the docking score of the ligands of one target against the docking score of the second one. In this plot (Figure 1), a ligand can be described by the coordinates or docking scores of both targets. Since they are coordinates, the ligand describes an *arrow* or vector, starting from the origin. The angle described by the vector is the selectivity; in fact, the formula of the tangent is the formula for selectivity (Equation 1).

(1) $$\begin{array}{l} \text{tan }\alpha \;=\frac{Target\;\text{\#2 }ligand's\;affinity}{Target\;\text{\#1 }ligand's\;affinity} \end{array}$$

Moreover, the magnitude of the vector is likely related to how potent the ligand is. Greater affinities reflect greater magnitudes. With the graph (Figure 1), there is a sense of what a multitarget drug would be: one that equally distributes its magnitude among the two targets. In other words, a ligand that had the same affinity for both targets. Measuring the multitargeticity of a dual ligand can be as simple as obtaining the tangent of the angle (Equation 1). If the tangent equals 1 (α = 45°), then mathematically, the ligand attacks both targets with the same “strength.” However, this interpretation was meant for more targets. In these scenarios, a single parameter for defining multitargeticity would not suffice because more angles are involved.

A radial plot was considered to extend the analysis to further dimensions (Figure 1). The central idea was kept: **a** multitarget ligand would equally distribute its *strength* among all the targets. In the radial representation, a multitarget drug would appear as a regular polygon, and the area could be considered the magnitude or strength of the ligand. Multitargeticity could be defined by the similarity between the figure described by the ligand and a regular polygon. We calculated some parameters that could be used to describe this relationship and therefore give a quantitative definition of multitargeticity. However, the area and shape are sensible to the order in which the targets are analyzed. A different order would give a completely different value, as shown in Figure 1.

The solution was to treat ligands as vectors and extend the analysis to further dimensions, even if it cannot be visualized. With two targets, an ideal multitarget drug is a vector that makes a perfect square in a 2D plane. In 3 dimensions, a cube would be the shape of an ideal multitarget ligand. Therefore, a hypercube is the central analysis of this interpretation. In contrast, a different distribution of affinities would produce the shape of a rectangle, rectangular cuboid, or hyperrectangle, depending on the number of targets analyzed. Measuring the similarity between the hyperrectangle and a hypercube is, in fact, a measure of multitargeticity, which is only a mathematical definition of how much a ligand equally distributes its *strength* among all the targets. With this analysis, another step was made toward defining and measuring multitargeticity.

One of the several complex diseases that can be treated with a multitarget drug approach is multiple sclerosis (MS). MS is a disease that affects the central nervous system. Currently, MS is the neurological disease with the highest incidence; in 2013, ~2.3 million people were estimated to have MS (Browne et al., 2014). The pathophysiology of the disease is based on the demyelination of axons, primarily due to the loss of oligodendrocytes, cells responsible for maintaining the myelin sheaths around them (Dobson and Giovannoni, 2019). Although the exact origin of MS is not known, it is well-established that it causes damage to the myelin sheath. Depending on the type of MS and the damage present, the slow transmission of electronic impulses may lead to axon loss, consequently damaging the optic nerve and leading to degeneration of vision, weakness, atrophy, and muscular rigidity, coordination and balance failures, recurrent fatigue, dysphagia, depression, and anxiety, among other symptoms (Huang et al., 2017). PAR-1 and KLK-6 are two molecules of biological interest as possible targets for MS treatment due to their role in oligodendrogliopathy and autoimmune response (Burda et al., 2013). PAR-1 is a protease-activated receptor involved in coagulation, angiogenesis, proinflammatory responses, oligodendrocyte death, and myelination (Macfarlane et al., 2001; Yoon et al., 2015; Pan et al., 2016; Lee et al., 2017). Antagonists of PAR-1 have been shown to reduce the symptoms of experimental autoimmune encephalomyelitis (EAE), which is the most studied animal model for MS (Kim et al., 2015). Kallikrein 6 (KLK-6) is the most abundant serine protease in the central nervous system (CNS) and has proteolytic activity against myelin basic protein (MBP) and amyloid precursor protein, which are part of the myelin sheath and are involved in myelination (Burda et al., 2013; Yoon and Scarisbrick, 2016). KLK-6 is also involved in T-cell survival and apoptotic signalizing (Scarisbrick et al., 2011).

Additionally, recently, a new drug for secondary progressive MS was approved by the FDA: siponimod, which is sold under the trade name Mayzent^®^. Siponimod is a dual drug itself: it binds to sphingosine-1-phosphate receptor 1 and 5 (S1PR1 and S1PR5) (O'Sullivan et al., 2016). Siponimod reduces oligodendrocyte death and demyelination, acting as an effective neuroprotective agent (Behrangi et al., 2019). S1PR1 is involved in regulating the inflammatory response and therefore is of interest as a third biological target (Chi and Nicol, 2010).

In this work, we present the construction of multitarget indexes as parameters that can define multitargeticity, their evaluation on several datasets, and their use in identifying potential multitarget ligands for PAR-1, KLK-6, and S1PR1.

## Materials and Methods

### Construction of an Experimental Training Set: Multi-Kinase Ligands

We selected 10 known FDA-approved drugs labeled multikinase-directed drugs as models of multitarget drugs (Li et al., 2016). Additionally, two non-multikinase drugs were included as negative controls. The binding affinity (defined in terms of K_i_) of each drug to its target was searched in the Binding DB (Gilson et al., 2016). The targets of each ligand were selected according to the FDA approved information. For the negative controls, tyrosine-protein kinase ABL1 was included in the analysis. The K_i_ was transformed into pM units and linearized. The objective of this analysis was to test if the multitarget index could correctly classify drugs using experimental values. The analysis was done, as stated in section Construction of the Multitarget Index.

### Construction of a Docking Training Set: Multi-Kinase Ligands

We selected 10 known FDA-approved drugs labeled multikinase-directed drugs as models of multitarget drugs (Li et al., 2016). The full list of the drugs we considered can be found in the Supplementary Information. Three non-multikinase drugs were also included as negative controls. The 13 ligands were docked on the tyrosine-protein kinase KIT (PDB id: 4HVS), vascular endothelial growth factor receptor 2 (PDB id: 3VO3) and platelet-derived growth factor receptor beta (PDB id: 1SHA). The docking studies were carried out in Molegro Virtual Docker version 6.0 using the standard protocol suggested by the manufacturer. All waters, cofactors, and non-active ligands were removed from the workspace. MolDock optimizer was used as the running algorithm with 25 runs per ligand. A sphere with a radius of 15 Å was constructed around the active sites of the three proteins and selected as the search site. The poses with the lowest MolDock score were used for further analysis. The objective of this analysis was to determine if the multitarget index could correctly classify drugs using theoretical values. The data were processed, as stated in section Construction of the Multitarget Index.

### Construction of an Experimental Evaluation Set: DUD

We downloaded the database of DUD (Directory of Useful Decoys; Huang et al., 2006), containing the energy scores of nearly 98258 molecules against 40 targets; some targets had a smaller number of calculated energies but were included in the analysis, as this would challenge the indexes. The package was cleaned so that only the negative energies of each ligand were analyzed. The objective of this analysis was to determine if the multitarget index could filter an extensive database. The data were processed, as stated in section Construction of the Multitarget Index.

### Virtual Library of Ligands for MS

#### Selection and Construction of the Virtual Library

**Figure 2:**
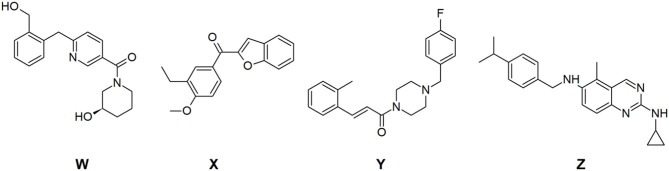
The four scaffolds used for ligand construction.

For ligand construction, PAR-1 antagonists with demonstrated activity were searched. Vorapaxar is a commercially available platelet antiaggregant whose mechanism of action is PAR-1 antagonism; therefore, it was used as a reference ligand. F16357 and SCH79797 are molecules whose antagonism has been previously studied, and thus, they were used as starting points for the design of multitarget molecules (Manaenko et al., 2013; Readmond and Wu, 2017). Four possible scaffolds were selected for PAR-1 (Figure 2), of which scaffolds W and X were obtained by scaffold hopping from F16357 and vorapaxar, respectively, with the help of Mcule (Kiss et al., 2012). F16357 was used as scaffold Y, and scaffold Z was an annular modification of SCH79797.

**Figure 3:**
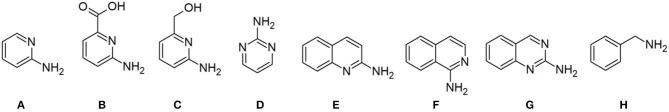
Proposed isosteres that may interact in the active site of KLK-6.

For the selection of KLK-6 ligands, benzamidine isosteres were designed, because this compound is known to be a serine protease inhibitor (Silva et al., 2017). The selected benzamidine isosteres were aminopyridine (A), aminopyridine with carboxylic acid (B), aminopyridine with alcohol (C), 2-aminopirimidine aminoquinoline (E), aminoisoquinoline (F), aminoquinazoline (G), and benzylamine (H) (Figure 3). Currently, there are no commercially available drugs whose mechanism of action is selective inhibition of KLK-6. However, it was found that 0HM, a benzylamine derivative, was previously determined as a compound with high binding energy to KLK-6 and thus was used as a reference ligand for this enzyme (Liang et al., 2012).

Finally, 211 compounds were constructed from a combination of both types of ligands with the help of Marvin Sketch 16.2.22.0 and saved in ^*^.smiles format. The three-dimensional geometry was optimized with Spartan '14 (1.1.4) using MMFF and HF 6-31 G^*^.

#### Molecular Docking

The crystalline structures of PAR-1 complexed with vorapaxar (PDB id: 3VW7), human S1PR1(PDB id: 3V2Y) and human KLK-6 with 0HM (PDB id: 4D8N) were downloaded (Bernett et al., 2002; Hanson et al., 2012; Liang et al., 2012; Zhang et al., 2012). The water molecules and co-crystallized ligands were removed from the work area. The docking procedure was carried out in the same manner used for multikinase ligands.

#### Cheminformatics Analysis

The smiles codes of the 211 ligands were placed in admetSAR in order to predict some of their pharmacokinetic properties (Cheng et al., 2012). The following probability scores were obtained: permeability of the blood-brain barrier (BBB), human intestinal absorption (HIA), glycoprotein P substrate (PGP-substrate), carcinogen, acute oral toxicity (AOT) and inhibition of hERG (human Ether-à-go-go-Related Gene). A coefficient of +1 was assigned to all values that fulfilled the following conditions: BBB +, HAI +, PGP-non-substrate, non-carcinogen, AOT III, or IV and weak hERG inhibitor. Otherwise, a negative coefficient was assigned in such a way that the desirable properties were considered positive. With these coefficients, an average of the chemoinformatic properties was calculated, which was called Chemoinformatic Score (CIS).

### Construction of the Multitarget Index

Vector analysis, mentioned in the introduction, was used as the mathematical basis for the index construction. Besides, vector analysis allowed new interpretations of ligands, concepts, and parameters that may have a significant impact on multitarget drug design.

#### Order of a Ligand

The core idea of the index is to interpret ligands as vectors. The theoretical affinity or score for a target may be interpreted as a coordinate within this vector. This interpretation treats targets as independent variables that are orthogonal to each other. The ligand (L) is then defined as follows:

(2) $$\begin{array}{l} \overset{⇀}{\text{L}}\;=\;\left({\text{a}}_{1},{\text{a}}_{2},\ldots ,{\text{a}}_{\text{i}}\right) \end{array}$$

where a_i_ is the affinity for each target. The usefulness is that the number of targets is now coded as the number of coordinates or dimensions. Therefore, the order of a ligand (n) relates to the number of targets being tested: a multitarget of order n.

#### Force of a Ligand

This parameter corresponds to the norm of the vector, which is a metric that combines all the affinities of the ligands into a single value. It is generally understood as magnitude, meaning that greater values correspond to ligands whose particular affinities are large. It is a useful parameter when trying to compare combined affinities. However, this metric is also sensible to extreme values. The force of each ligand (F) was calculated in the following way:

(3) $$\begin{array}{l} \text{F }=\;\overset{⇀}{\left\|\text{L}\right\|}\;=\;\sqrt{{a_{1}}^{2}+{a_{2}}^{2}+\ldots +{a_{n}}^{2}} \end{array}$$

Plotting the ligand can enhance the interpretation, as seen in Figure 1. However, for more than three coordinates, representations must be truncated into a radial web for better visualization.

#### Binding Capacity and Total Multitarget Capacity

Because each coordinate is a vector, the cross product of all the targets will give a new vector. This new vector is an indirect measure of the binding capacity of a ligand, which geometrically corresponds to an nth volume (Equation 4). This metric is more sensible than the force because it is a multiplication of affinities, and considerable differences between affinities have greater repercussions. This operation is the same as calculating the geometric mean. We interpreted the metric as the binding capacity, a measure of a ligand's tendency to bind to more targets. Higher binding capacity means it can bind efficiently to more targets.

(4) $$\begin{array}{l} \text{Bc}=\text{geometric mean}=\sqrt[{\text{n}}]{\underset{\text{i}=1}{\overset{\text{n}}{\prod }}{\text{a}}_{\text{n}}} \end{array}$$

The average is interpreted as a ligand's affinity to all the targets. The average simulates a drug that has equal affinities for all the proteins. Therefore, the average (μ) is defined as the total multitarget capacity of the ligand (MTc) (Equation 5). It is only a capacity since it is an idealized value.

(5) $$\begin{array}{l} \text{MTc}=\mu =\frac{1}{\text{n}}\overset{\text{n}}{\underset{\text{i}=\text{n}}{\sum }}{\text{a}}_{\text{i}} \end{array}$$

Finally, the quotient of the binding capacity and the total multitarget capacity gives a proportion of how much of that multitarget capacity is being used. If the binding capacity equals the total multitarget capacity, then the ligand is a true multitarget ligand. By itself, this quotient is an index of “multitargeticity” that can be expressed in percentage for easier reading and read as “used multitarget capacity” (U_MTc_).

(6) $$\begin{array}{l} {\text{U}}_{\text{MTc}}=\frac{\text{Bc}}{\text{MTc}} \end{array}$$

#### Index Standardization, Definition, and Interpretation

Since the idea behind the index is to compare different ligands, it is necessary to standardize the index. The following scheme was proposed: the ligand should be its own reference for standardization. This can be achieved through the following formula, in which the individual contribution of each affinity to the force is calculated.

(7) $$\begin{array}{l} \hat{{\text{L}}_{\text{s}}}=\text{n}{\left(\frac{\overset{⇀}{\text{L}}}{\text{F}}\right)}^{2}=\left(\text{n}{\cdot \left[\frac{{\text{a}}_{1}}{\text{F}}\right]}^{2},\text{n}{\cdot \left[\frac{{\text{a}}_{2}}{\text{F}}\right]}^{2},\ldots ,\text{n}{\cdot \left[\frac{{\text{a}}_{\text{n}}}{\text{F}}\right]}^{2}\right) \end{array}$$

where n is the number of targets, F is the norm or force of the ligand, and a is the affinity. This formula also standardizes the mean, or multitarget capacity (MTc) to 1, independently of the number of targets or the type of input used. For simplicity, the new coordinates were renamed “standardized affinities” $\left(\hat{\text{a}}\right)$.

(8) $$\begin{array}{l} \hat{{\text{L}}_{\text{s}}}=\left(\hat{{\text{a}}_{\text{a}}},\hat{{\text{a}}_{2}},\;\ldots ,\;\hat{{\text{a}}_{\text{n}}}\right) \end{array}$$

This simplifies the standardized, used multitarget capacity (U_MTc_) to a simple geometric mean ranging from 0 to 1, effectively making it an index or measurement of “multitargeticity.” As in linear regression, a quadratic estimator exacerbates the value, making it ideal for a multitarget index (Equation 9).

(9) M1stTi=(∏i=1na^nn )2=(∏i=1na^n)2/n

The interpretation is the one originally described in the introduction: how similar the hyperrectangle described by the ligand is similar to a hypercube. Alternatively, in a less abstract way, it is an efficiency measurement: how much “multitarget capacity” is being used.

#### A Second Multitarget Index

A second parameter was calculated; the standard deviation (σ). With this, another index was constructed that could measure the dispersion of the affinities: the bigger the value, the less variation among the targets. Since the standardized affinities' mean equals one, the second multitarget index is defined as follows:

(10) MTi2nd=1−σ

As in ^1st^MTi, the value can be expressed as a percentage (%). This is a more sensitive parameter that ranges from 1 to negative values. This index also encodes selectivity: smaller values, even negative, indicate more selectivity.

#### Defining of a Multitarget Ligand

With the two indexes, we propose the following values for classifying a ligand as a multitarget:

$$\begin{array}{l} \text{The ligand is multitarget if}{\;}^{1\text{st}}\text{MTi }\geq \;0.84\text{ and}{\;}^{2\text{nd}}\text{MTi }\geq \;0.60 \end{array}$$

These values correspond to an ~20% deviation from the mean affinity. It is worth mentioning that, although this gives a quantitative definition of multitargeticity, ligands that do not fulfill the criteria should not be discarded. These indexes quantify the dispersion and variation of the affinities and do not indicate in any way the potency.

#### Multitarget Potency and Selectivity

Equally low affinities will give high MT indexes values. For this reason, a final critical parameter was introduced: the multitarget potency. This value is the product of the force times, both multitarget indexes (Equation 11), which is the equivalent of calculating how much of that force is due to the multitargeticity of the ligand.

(11) $$\begin{array}{l} {\text{P}}_{\text{MT}}=\frac{\text{F}}{\sqrt{\text{n}}}\cdot^{1\text{st}}\text{MTi}\cdot^{2\text{nd}}\text{MTi} \end{array}$$

We propose the multitarget potency as a metric for drug design since the highest values of potency represent a possible multitarget hit or even multitarget lead. In the same line of thought, the next parameter that we propose is an attempt to identify selective ligands. The selectivity is calculated as follows:

(12) $$\begin{array}{l} \text{S}=\frac{\text{F}}{\sqrt{n}}\cdot \left(1{-}^{1\text{st}}\text{MTi}\right)\left(1{-}^{2\text{nd}}\text{MTi}\right) \end{array}$$

Both parameters maintain the desired properties (higher values indicate higher potency and selectivity) and are useful for identifying possible multitarget and selective ligands. These metrics, as with any other, have their benefits and drawbacks and will be discussed further on.

## Results and Discussions

### Performance of the MT Indexes in the Experimental Training Set

With the criteria set on point 2.5.6, of the 10 approved multikinase drugs analyzed (imatinib, sunitinib, dasatinib, afatinib, bosutinib, lapatinib, nintedanib, pazopanib, sorafenib, vandetanib), only afatanib was not classified as a multitarget ligand. Although it is biologically active in both of its targets, the epidermal growth factor receptor (EGFR) and the receptor tyrosine-protein kinase erbB-2 (HER2 or erbB2), it has a considerable preference over EGFR (K_i_= 0.1 vs. K_i_ = 5 [nM]).

The two negative controls did not fulfill the criteria to be cataloged as multitarget drugs. In fact, the epidermal growth factor receptor (EGFR) is the main and only target for gefitinib and erlotinib (Wishart et al., 2018). The indexes can reliably classify and discriminate multitarget molecules in experimental values, giving strength to the analysis.

**Figure 4:**
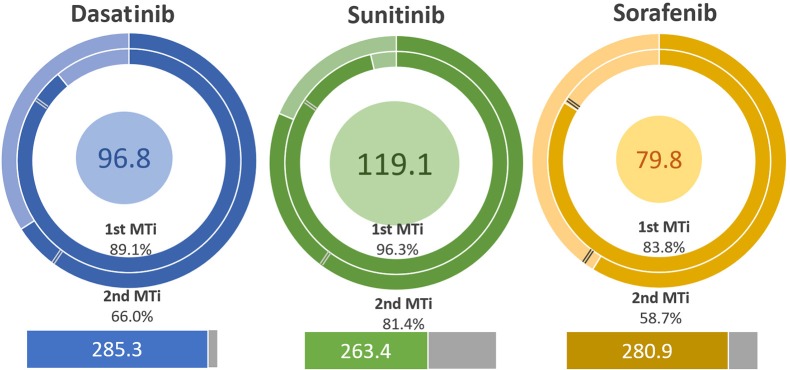
Dasatinib had 3 targets (*n* = 3) tested and greater affinities toward those, while sunitinib had order *n* = 5 and gefitinib *n* = 4. That is why, although sunitinib has better MTi values, dasatinib has more multitarget potency (the number inside the circle). All the values are presented as % (or times 100). The threshold for considering a ligand multitarget is viewed as a cut in the circles. The inner and outer rings are the 1st and 2nd MT indexes, respectively.

The multitarget potency, the critical parameter proposed, supports the findings, making dasatinib the most potent multitarget drug of the analyzed set (P_MT_ = 16.4). Sunitinib was de 2nd most potent multitarget (P_MT_ = 14.6); although it had better indexes, the total strength was reduced since it had more targets tested, and the affinities were not as strong as dasatinib (Figure 4). In contrast, gefitinib was the least potent (P_MT_ = 14.6), but it was also the 2nd most selective ligand of the dataset (S_gefitinib_ = 2.7 vs. S_dasatinin_ = 1.3 and S_sunitinib_ = 0.2), being afatinib the first (S_afatinib_ = 2.9).

^*^All P_MT_ and S values are dimensionless.

### Performance of the MT Indexes in the Docking Training Set

**Figure 5:**
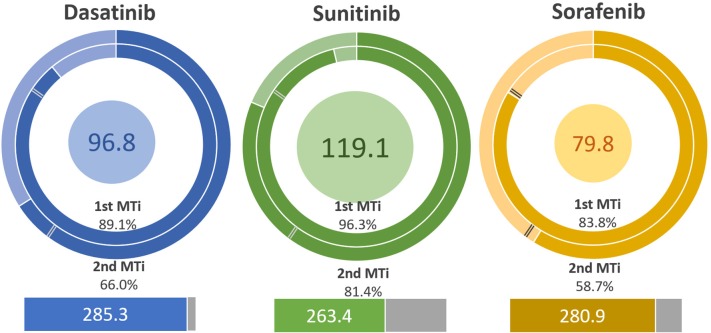
Comparing with Figure 4, the multitarget tendency of sunitinib and dasatinib remains similar. The difference in the calculated affinities gives the change in the potency. Sorafenib is an example of how the MT indexes affect the strength of the ligand, since that strength is unevenly distributed. The inner circle is the MT potency. The inner and outer rings are the 1st and 2nd MT index, respectively. The inferior rectangle is the force of the ligand.

With the criteria set on point 2.5.6, of the ten known approved multikinase drugs tested, only sorafenib was not classified as a multitarget ligand. By contrast, the three negative controls were classified as multitarget ligands according to the index. The apparent discrepancy between these results and the experimental ones is explained by considering that all the 13 ligands were docked in the same three targets, indistinctively if they were active or not, while in the experimental analysis, the preferred targets were analyzed according to each ligand. Sorafenib had larger calculated affinities than most of the ligands but was further apart from each other, which lead to it nor being classified as multitarget. The performance of sunitinib and dasatinib is observed in Figure 5, and the results agree with the experimental set. The only difference is that sunitinib is, in this case, the most potent multitarget ligand.

The analysis still proves useful once the ligands are arranged in order of highest to lowest multitarget potency, or the force of each ligand is compared. It is important to emphasize that the purpose of the index is not to reclassify drugs but instead to provide useful metrics for analyzing data and aiding in the drug design process, especially in the design of multitarget drugs. In this case, seven multitarget ligands would be discovered or tested before encountering a non-multitarget drug previously classified as an MT drug. The multitarget indexes are useful when they are used with the force of the ligand. The top 7 ligands are indeed classified as multitarget ligands and are approved by the FDA as multikinase drugs, and a summary of the performance can be reviewed in Table 1.

**Table 1:** Sunitinib, imatinib, and sorafenib are approved multitarget drugs by the FDA.

**Name**	**4HVS MolDock score**	**3VO3 MolDock score**	**1SHA MolDock score**	**F**	**^**1st**^MT index (%)**	**^**2nd**^MT index (%)**	**P_**MT**_**
Sunitinib	−166.0	−131.5	−156.6	263.4	96.3	81.4	**119.1**
Imatinib	−193.9	−130.7	−163.2	285.1	90.4	69.1	**102.8**
Idelalisib	−138.7	−104.8	−129.5	216.8	94.7	77.9	**92.4**
Sorafenib	−201.6	−119.8	−154.8	280.9	83.8	58.7	**79.8**
Letrozole	−137.9	−98.4	−114.8	204.6	92.7	72.4	**79.3**

*Sorafenib did classify as a multitarget drug. However, the multitarget potency of sunitinib and imatinib compared to letrozole and idelasib (negative controls in purple) is greater. The rest of the molecules can be found in the Supplementary Information. The bold values indicate the main or critical parameter(s) being evaluated*.

Since this is an *in silico* evaluation, the scoring is affected by the computational limitations of the docking procedures. These limitations should be taken into consideration when applying the metrics previously described. These are virtual metrics and are sensible to the *in silico* scoring functions, which themselves do not reflect the *in vivo* effect. Furthermore, the indexes and metrics should be tested with experimental values to prove the strength, robustness, and validity of this classification. As mentioned above, the criteria for classifying a ligand as a multitarget can be modified, but the measure of multitargeticity persists.

### DUD Database

**Graph 1:**
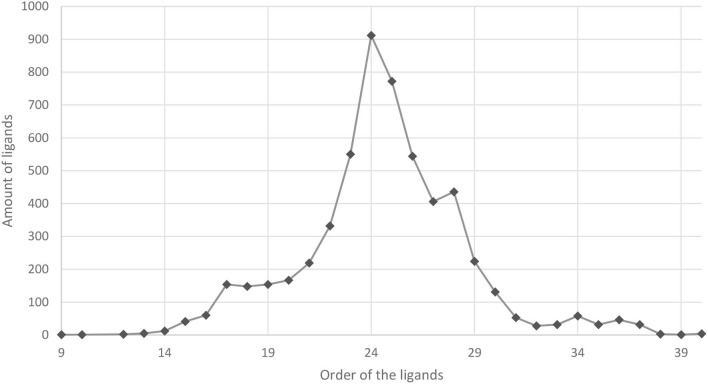
Distribution of how many orders of ligands were among the 5561 ligands identifies as multitarget.

From the 98258 ligands analyzed, 5561 molecules were found to be multitarget. This corresponds to about 5.7% of all the analyzed ligands. The orders of the ligands varied widely, ranging from 9 to 40. A total of 912 ligands were found to be multitarget of order 24. The distribution can be seen in Graph 1. The multitarget index filtered the ligands, meaning it does not classify every ligand as multitarget, and only a subset is eligible according to the criteria. It is important to notice that the chemical structures in this dataset were diverse. In these cases, the MT indexes gain strength as they “clean” the database and facilitating further research.

**Figure 6:**
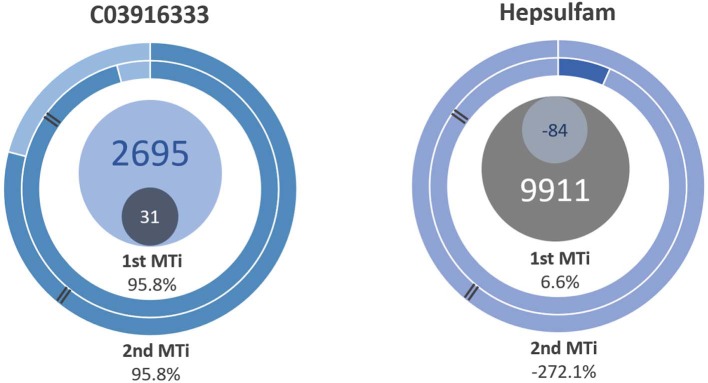
Multitarget parameters of the most potent multitarget ligand and hepsulfam, the 3rd most selective. The darker circle represents the selectivity, while the lighter represents de potency. Hepsulfam had a negative MT potency.

The most potent multitarget ligands were an unnamed compound of the ZINC database, which could be further tested to determine if its potential multitargeticity is only theoretical. This is the purpose of the indexes, to be useful in drug design and in identifying potential multitarget ligands. The selectivity was also used to identify the most selective ligands. In the DUD database, the third most selective ligand corresponded to hepsulfam, with the following MT indexes: 6.6% (1^st^) and −272.1% (2^nd^). A comparison is made in Figure 6. The selectivity was toward catechol-O-methyltransferase (COMT). Hepsulfam is an alkylsulfonate alkylating drug-like busulfan used in cancer therapy. COMT is a modulator of the dopaminergic and adrenergic response, and it can influence nausea and vomiting (Gan and Habib, 2016). Hepsulfam binding to COMT could explain mild nausea and vomiting seen in clinical trials, contrasting serious nausea present in other types of anticancer drugs (Ravdin et al., 1991).

### Advantages and Limitations of the Multitarget Indexes

The usefulness of using a multitarget index varies highly according to the necessities of each research group. As a first instance, the multitarget indexes give an initial quantitative and workable definition for what a multitarget drug is. They define and measure multitargeticity. The primary purpose of the analysis is in drug design for identification of *in silico* and potential *in vivo* multitarget drugs. In a sense, these indexes could be useful in identifying multitarget hits and leads in the drug discovery process. Second, because it is an index, it can be useful in data analysis when comparing several ligands or targets at the same time. Moreover, the analysis assumes that the targets are independent of each other, which provides freedom regarding the number of studied targets.

The index is also modifiable and perfectible in several ways. For example, if highlighting a particular target is desired, then coefficients can be introduced so that the affinities are weighted. More calculations may be performed on the affinities in previous steps without changing the procedure or interpretation of the index, such as introducing ligand efficiency metrics. The multitarget indexes do not only identify multitarget ligands but are useful when selectivity is desired, making them not only applicable in multitarget drug design but also in designing selective single-target drugs and in the drug discovery process in general. Finally, the analysis can be further perfected with more statistical rigor, more meaningful parameters, and an *in vitro* and *in vivo* extensions.

Like all other metrics, it has limitations that skew or simplify the underlying mechanisms. For example, equal affinities may not necessarily imply multitarget *in vivo* effectiveness, since there are more variables to consider.

Various suppositions are needed in order to treat ligands as vectors. The most obvious one is that indexes do not consider how the ligand binds the target or the mechanism of action. Second, it is assumed the affinities are calculated or measured under the same conditions. Third, although the coordinates can be any type of input, the final MT index value changes if the units of the affinities introduced are different; therefore, MT index values can only be compared if the data is processed the same way and is in the same units.

The performance of the MT indexes in all the training sets shows that they correctly classify drugs using experimental values, identify potential multitarget ligands *in silico*, and can filter an extensive database, making them valuable for the intended purposes.

### MT Indexes in the Virtual Library of Candidates for MS

With the known limitations and advantages, the MT indexes were used to analyze the experimental set. The 214 docked ligands were submitted to the analysis previously described and ranked in descending order of multitarget potency. Of the 211 designed compounds, 45 were derivatives from scaffold W, 34 from scaffold X, 29 from scaffold Y, and 103 from scaffold Z. Scaffold Z was present in 9 out of the top 10 most potent multitarget ligands. Benzamidine isostere A was present in 5 of them, hinting that an aminopyridine fragment may be ideal for a multitarget effect.

In most cases, a linker of 1 carbon atom and an ester group were found in the most potent ligands. More linkers diminished the MT index value, increasing the selectivity toward KLK-6, the only exception to this rule was the top, most potent molecule with four linkers. The reference molecules and their multitarget parameters can be seen in Table 2. In Table 3, the top 5 most potent multitarget ligands from the experimental set are presented.

**Table 2:** Summary of the multitarget metrics of the reference ligands.

**Name**	**KLK-6 MolDock Score**	**PAR-1 MolDock Score**	**S1RP1 MolDock Score**	**F**	**^**1st**^MT index (%)**	**^**2nd**^MT index (%)**	**P_**MT**_**
Vorapaxar	−183.0	−147.7	−128.2	267.9	91.7	70.2	**99.6**
Siponimod	−143.9	−184.2	−155.8	280.9	95.7	78.8	**122.3**
0HM	−185.5	−140.5	−148.2	275.9	94.1	74.5	**111.8**

*The bold values indicate the main or critical parameter(s) being evaluated*.

**Table 3:** Summary of the top 5 ligands which had the highest potency.

**Name**	**KLK-6 MolDock score**	**PAR-1 MolDock score**	**S1RP1 MolDock score**	**F**	**^**1st**^MT index (%)**	**^**2nd**^MT index (%)**	**P_**MT**_**
Z_b0_A_d4_	−210.2	−174.8	−177.9	326.2	97.2	82.6	261.9
Z_b0_C_0l1_	−193.4	−161.3	−188.9	314.8	97.5	84.8	260.5
Z_c0_B_0l1_	−190.1	−160.9	−176.8	305.4	98.2	86.6	259.5
Z_d0_A_p2_	−167.1	−153.4	−165.5	280.8	99.4	92.6	258.5
Z_b0_A_d1_	−202.5	−164.5	−183.8	319.1	97.2	83.2	257.9

**Graph 2:**
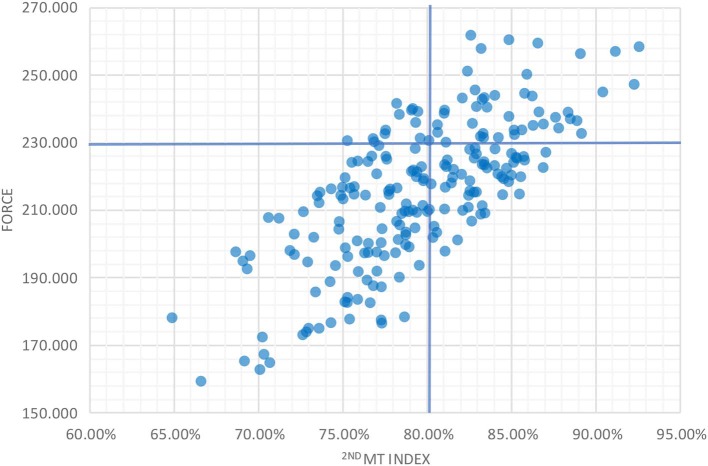
When plotted, the upper left quadrant is the most valuable or with the most potentially multitarget ligands.

In multitarget drug design, the multitarget potency combines the two indexes and the force. For choosing candidates, the force against the 2nd MT index can be plotted, and the regions divided into quadrants. The most favorable zone would be the upper left since it would group the most potent and most specific multitarget ligands (Graph 2).

### Chemoinformatic Analysis

With the help of the chemoinformatic score (CIS), the ligands were classified into three arbitrary categories: preferred (CIS> 0.75), sufficient (0.75> CIS> 0.5) and risky (CIS <0.5). In total, nine ligands (4.23%) entered the preferred classification. Of these, eight belonged to scaffold X, and 1 to scaffold Z. Overall, 165 ligands (77.46%) fit into the sufficient category, and 39 (18.31%) were classified as risky (see Table 4).

**Table 4:** Distribution of the chemoinformatic score among the ligands with the 4 scaffolds.

	**Preferred (CIS>0.75)**	**Sufficient (0.75>CIS>0.5)**	**Risky (CIS <0.5)**	**Total**
Scaffold W	-	31 (68.9%)	14 (31.1%)	45
Scaffold X	8 (23.2%)	15 (44.1%)	11 (32.3%)	34
Scaffold Y	-	22 (75.8%)	7 (24.1%)	29
Scaffold Z	1 (~1%)	95 (92.2%)	7 (6.8%)	103
Total	9	163	39	211

*Ligands with scaffold X were the most preferred in terms of pharmacokinetics theoretical properties*.

From the 45 scaffold W ligands, 14 (31.11%) were considered risky, while 31 ligands (68.89%) were considered sufficient; none reached a CIS> 0.75. From scaffold X, 11 ligands (32.35%) obtained a risky score, 15 ligands (44.11%) were sufficient, and 8 (23.23%) were preferred. Scaffold Y had seven ligands (24.14%) classified as risky, and 22 ligands (75.86%) classified as sufficient. Finally, seven ligands (6.80%) of scaffold Z were considered risky, while 95 ligands (92.22%) were considered sufficient, and only 1 (~1%) was preferred. These results can be seen in Table 5.

**Table 5:** Distribution of the chemoinformatic score among benzamidine isosteres.

	**Preferred (CIS>0.75)**	**Sufficient (0.75>CIS>0.5)**	**Risky (CIS <0.5)**	**Total**
Isostere A	8 (5.9%)	114 (83.8%)	14 (10.3%)	136
Isostere B	-	2 (15.4%)	11(84.6%)	13
Isostere C	-	7 (53.8%)	6 (46.1%)	13
Isostere D	-	6 (66.6%)	3 (33.3%)	9
Isostere E	-	10 (100%)	-	10
Isostere F	-	9 (100%)	-	9
Isostere G	1 (10%)	7 (70%)	2 (20%)	10
Isostere H	-	9 (81.8%)	2 (18.2%)	11

*A considerable proportion of ligands with isosteres B and C were classified as risky, while ligands with isostere A had more preferred CIS*.

These results show that scaffold Z has the most balanced theoretical pharmacological properties. However, scaffold X also presented desirable properties in the CIS; scaffold X is pharmacokinetically desired. It is also worth mentioning that the aminopyridine derivatives with carboxylic acids (B) and alcohols (C) presented a risky CIS in the chemoinformatic analysis. Therefore, ligands with these isosteres are not considered candidates for therapeutic applications.

### Pharmacokinetic and Pharmacodynamic Viable Candidates

**Figure 7:**
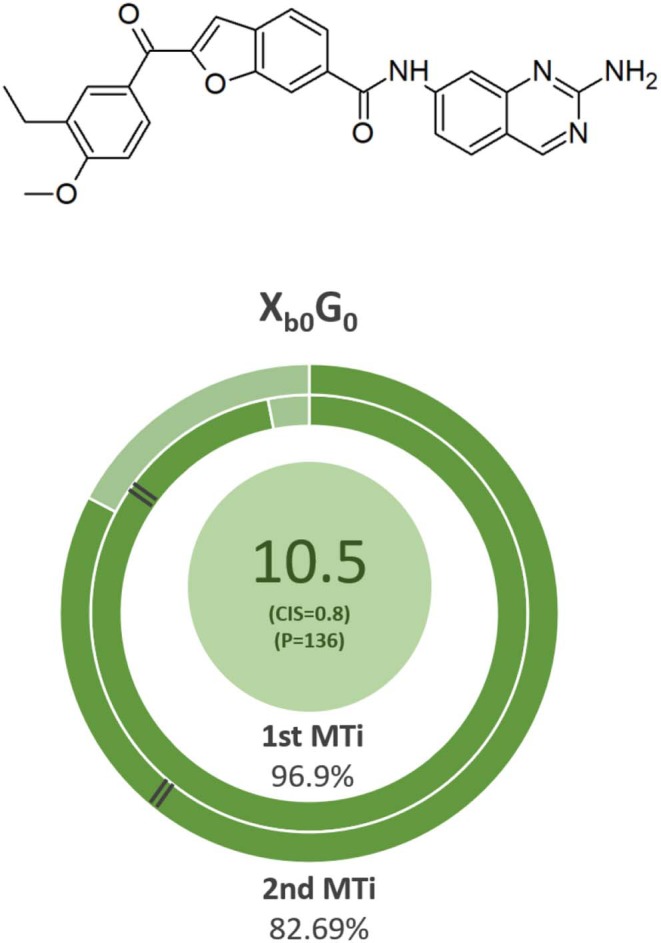
Ligand Xb0G0 had the highest combined multitarget theoretical affinity and *in silico* ADME profile. The parameters of the MT indexes analysis. The final score is the combination of the MT potency and CIS (in parenthesis).

For determining possible final candidates, the CIS score and multitarget potency were combined with the geometric mean. The final table (Table 6) groups the ligands that combine the highest potency and CIS values, meaning they are the most likely to have a biological effect while remaining relatively safe. This is a theoretical approach; therefore, the top molecules are potential multitarget alternative candidates to treat MS. The ligands with the highest combined score shared scaffold X in most of the cases (10 out of the 20 top ligands), with scaffold Z being the second most shared among the top 20. In 9 out of the top 10 ligands, isostere A was present in the ligand structure. It is also noted that a small linker is optimal for joining these two fragments. From these results, it is assumed that isostere A, as well as scaffolds X and Z, contribute to theoretical multitarget effects. However, scaffold X ligands do not have as much multitargeticity nor force as ligands with scaffold Z but remain the safest and more pharmacokinetically favorable. The applied multitarget metrics simplified the analysis and criteria for determining viable candidates. In Figure 7, the most viable candidate is presented.

**Table 6:** Summary of the top 5 ligands which had the highest combined values of Potency and CIS.

**Name**	**F**	**^**1st**^ MTi (%)**	**^**2nd**^MTi (%)**	**P**	**CIS**	**Final value**
X_b0_G_0_	**294.1**	96.9	82.7	235.8	0.81	**10.5**
Z_b0_A_s0_	**314.3**	95.8	79.4	239.3	0.78	**10.4**
X_b0_A_s0_	**269.4**	97.8	85.7	225.9	0.82	**10.4**
X_b0_A_m1_	**273.4**	97.2	84.0	223.3	0.82	**10.3**
X_b0_A_m0_	**261.1**	98.2	86.9	222.7	0.80	**10.1**

*The bold values indicate the main or critical parameter(s) being evaluated*.

## Conclusions

As multitarget drugs are designed and tested, methods for effectively comparing and optimizing ligands are required. We present a new interpretation of ligands as vectors; new multitarget definitions and metrics, such as order of a ligand, the force of a ligand, binding capacity and multitarget capacity; and two multitarget indexes representing multitarget potency and selectivity, all of which might prove useful in drug design. The training sets allowed the identification of the advantages and disadvantages of using these metrics in multitarget drug discovery. The data analyzed through the MT indexes served to identify pharmacokinetically and pharmacodynamically viable multitarget therapeutic candidates for MS. The indexes were also useful for identifying selective ligands. The definitions, metrics, and analysis proposed here may provide a guide toward the definition of “multitargeticity.”

## Data Availability Statement

All datasets generated for this study are included in the article/Supplementary Material.

## Author Contributions

All authors have contributed significantly to this work, in the design of the model, performance of the computational experiments, analysis of the generated data, and writing the paper. All authors read, edited, and approved the final manuscript.

### Conflict of Interest

JS-R was employed by company Ciencia y Estrategia S.A. de C.V. The remaining authors declare that the research was conducted in the absence of any commercial or financial relationships that could be construed as a potential conflict of interest.
